# Dental Items

**Published:** 1871-06

**Authors:** 


*Dental Items.*—We have received another beautiful specimen of calcified pulp, from Drs. Story and Watkins, of Eutaw, Alabama, which, as Dr. S. writes, "still retains its position in the cavity, with the roots united by the depositing of the same osseous matter—this I have often seen before but the pulp in a similar condition never—and it may prove a subject of much interest."

We shall soon have a large and beautiful collection of this pathological condition of tooth-pulp in the museum of the Baltimore College, and feel very grateful to our friends for their kindness in forwarding interesting specimens of dental anomalies.

In the "Dental Office and Laboratory" Dr. W. C. Wren gives the following method of working Pyroxyline Base:

"You get up dies the same as you would for a metal plate. The only extra tools you require is a press—an ordinary letter press will do.

"Having your dies made, you heat them, and the best way to heat them is to put them in boiling water. Then place the plate of pyroxylene between the dies and swage it home in your press. You may split some plates at first, but a short experience will teach you. After you have swaged the plate home, you want to let it remain in the press until cold ; then take out and trim to size and shape.

"The pyroxylene plate can be altered or bent slightly with a pair of plyers. just the same as a gold or silver plate.

"After you have fitted to the mouth and ground the teeth to it, you take a quantity of filings of the material and place the filings around the teeth outside where the band would come on a gold plate. The filings should be wetted with a solution composed of two parts ether and one part alcohol, which will dissolve them, and unite them to the plate. Put a quantity on the outside, if too much, you can easy file off when hard. As soon as the filings are put on the outside, cover up with plaster to keep the teeth in place. When the plaster is hard, remove the wax perfectly. It can be done by putting the work into clean boiling water. After the wax is thoroughly removed, place the filings around the pins of the teeth. Put a little on at a time, being careful to separate as it dries between each pin, and from the plate, so that the filings as it shrinks, will cling to the pin. After the pins are covered and dry, commence to fill any spaces there may be between the plate and teeth. Do it gradually, and push it down hard with an instrument. Keep filling until you have as much material as you want around the inside of the gums. When it is thoroughly dry, you can scrape and polish if necessary. If your dies have been smooth, and you have manipulated right, no polishing is necessary.

Keep the plate on the plaster until you put it in the mouth.

The best way to make the filings is to take the pieces you cut off the plate, and file with a coarse file as you want them.

The material is very light, and as the saying goes: "an ounce makes a pound."

In the *Canada Journal of Dental Science*, Dr. Beers gives the following account of inferior central incisor teeth at birth:

"The teeth were to all appearances as perfect in shape as temporary incisors ever are, and abundantly supplied with good enamel. As might be expected, they were slightly loose, but had the feeling under the finger, and, from what could be determined by observing the gum opposite them, of having some attachment to the socket; and I felt assured that the fangs were formed, though not fully, and that the original fibrous septa had ossified and become sufficiently firm and high, to give comparative solidity to the teeth. I hardly supposed that the fangs were as *fully* formed as they would be at the normal period of eruption;—that they had shared in the premature calcification and development of the crowns; because the cementum is the last tissue of the tooth formed. On the other hand, the question arose as to whether or not the calcification and growth of the whole tooth had not been facilitated by the early emergence of the crown through the gum; and again, if it might not be possible that the premature eruption of the crown was actually by a premature calcification of the fang, as the eruption at the regular period is generally believed to be the consequence of the increasing size of the fang pressing the tooth upwards, and absorbing the gums. As a rule, the cellular tissue or gums of the child are firm, and of a cartilaginous consistence at time of birth, and the eruption of the teeth at the seventh month is attended with various slight and critical derangements, either a direct consequence of this normal physiological developement, or connected with the irritation common about the time of the protrusion. Now, of course, there must be considerably greater resistance in the gums at the seventh month, than in embryo, and the counter pressure exerted upon the incompletely ossified pulp would excite a degree of irritation, which would be altogether absent and avoided in premature eruption; and the sympathetic excitement of the emergence of the lower incisors, at the tenderest age, would be escaped by the child, to its advantage, I believe. Here, in the case I describe, the saccular stage had terminated and the eruptive had occurred when the child was in embryo, whereas, according to the normal period, the teeth should at this time have been in the saccular stage, and the eruptive should not have taken place until about the seventh month. The teeth were allowed to remain, and are now perfectly firm and wholly erupted, a simple cap of gutta-percha, underneath, of which was tied a piece of rubber tubing, preventing the mother's breast from suffering from their pressure while the child was suckling."

Cases of swallowing artificial teeth on plates, are becoming quite common in England. The "London Lancet" of May 13, contains an article from Dr. Jno. Matthews, which is of interest as the details are given of the the operation for the removal of the artificial teeth from the œsophagus:

On the night Sunday March 26th last, I was called up at 1.30 A. M. to see Mrs G. J.——The messenger only waited to say that she was choking and ran off On arriving at the house, I was informed that the family had been aroused by the screams of the sufferer, who was liable to frequent epileptic fits. On reaching the bedside, she was found clutching at her throat, laboring for breath, and partially unconscious, having evidently just had a severe attack. She could not swallow, nor speak much above a whisper. It was then observed that a plate holding six artificial teeth, known to be in her mouth when she went to rest, had disappeared. As it could not be found anywhere, the inference naturally arose that she had swallowed it during her fit; but of this we were uncertain, since she often misplaced or hid articles of dress, ornaments, &c., during the half consciousness succeeding her attacks.

I immediately introduced a finger as far as possible down the throat, but could feel nothing, although it reached below the glottis. The patient was so much distressed by this that I only repeated it once with a finger of the other hand; but to no purpose, nor could any prominence be felt externally. In the absence, therefore, of absolute proof that she had swallowed the teeth, I thought it might be an aggravated case of histeria or epileptic spasm of the pharynx; and having prescribed accordingly, left her for a few hours, enjoining a more careful search for the missing teeth. On visiting her at 10 A. M. on Monday, I found matters *in statu quo*. She had not been able to swallow either food or medicine; but the dyspnœa was not so severe. I then explored with both fingers; but to no purpose, and left, expecting that if the plate were there it would slowly find its way into the stomach. At 2 P. M. no alteration; teeth not found. I then proposed a consultation with Mr. Holmes Coote; and he accordingly saw her at 6.30 P. M. He also was in considerable doubt as to whether the teeth were there or not, since he could not feel them either by the fingers or a pair of long forceps which he brought with him. I may add that our efforts were seriously impeded by the streuous resistance of the sufferer, especially with Mr. Coote; and she finally declared that she would not suffer him to touch her any more, otherwise I have no doubt that he would have relieved her. We then left, having directed a dose of castor oil to be given in the morning.

On Tuesday morning I visited her. The castor oil ordered could not be taken, and she was still in so much distress that, after a gentle remonstrance, she promised to be more submissive to further exploration. This I attempted still with the finger, feeling sure that the obstruction was within its reach, if her description of her suffering were accurate as to place. On this occasion the left finger went further than before, reaching about an inch below the glottis (I had previously used the right finger without success); and I then had the satisfaction of feeling with my nail the edge of the plate. The patient was gasping for breath during this proceeding; I therefore removed my finger, and after a brief interval reintroduced it, sliding down at the same time, close to it, a pair of polypus forceps, by which I was able to grasp the thin edge of the plate, which was lying with its long axis transversely, its hollow closely applied to the back of the trachea, the teeth downwards. Finding it immovable except by much force, and unwilling to risk laceration of the œsophagus, after another brief interval I once more inserted the left finger and forceps, grasped the plate, and then slid the forceps to its other end, so as to tilt it by the aid of the finger, when it was at once easily extracted. All the symptoms were of course immediately relieved. There was very little, in fact scarcely any, bleeding; and in two days the patient was as well as usual.

I have been asked why I could not at once feel the plate with the finger, I did not explore with a bougie or a probang. My reply is—1st, that I never use an instrument when there is the least chance of a finger being successful; 2nd, that I had reason, as I have before said, to believe that it would prove to be within reach when the sufferer was quieter; 3d, that I always found the glottis so open that I should very likely have pushed such an instrument into it; and, lastly, I did not like to run the risk of pushing the plate down into the stomach. I cannot help thinking this last likely to be a very dangerous event or proceeeding, not only from the probability of laceration of the œsophagus by the angles and edges of so large a foreign body, but still more from the chance of its producing a fatal intestinal obstruction, not to speak of the very great anxiety of mind inflicted on all concerned until it shall have passed, if it eventually do.

I think we may learn from this case not to give up an apparently hopeless effort too hastily, and that epileptics should not wear artificial teeth when going to sleep or at night, or indeed at any other time, unless properly secured from displacement.

